# Development and pilot of an international survey: ‘Radiation Therapists and Psychosocial Support’

**DOI:** 10.1002/jmrs.286

**Published:** 2018-06-07

**Authors:** Kelly L. Elsner, Diana Naehrig, Georgia K. B. Halkett, Haryana M. Dhillon

**Affiliations:** ^1^ Sydney Medical School University of Sydney Sydney New South Wales Australia; ^2^ School of Nursing, Midwifery and Paramedicine Faculty of Health Sciences Curtin University Perth Western Australia Australia; ^3^ Centre for Medical Psychology and Evidence‐based Decision‐making, School of Psychology University of Sydney Sydney New South Wales Australia

**Keywords:** Patient anxiety, pilot survey, psychosocial support, radiation therapists, survey development

## Abstract

**Introduction:**

Up to one third of radiation therapy patients are reported to have unmet psychosocial needs. Radiation therapists (RTs) have daily contact with patients and can provide daily psychosocial support to reduce patient anxiety, fear and loneliness. However, RTs vary in their values, skills, training, knowledge and involvement in providing psychosocial support.

The aims of this study were to: (1) develop an online survey instrument to explore RT values, skills, training and knowledge regarding patient anxiety and psychosocial support, and (2) pilot the instrument with RT professionals to assess content validity, functionality and length.

**Method:**

An online cross‐sectional survey, titled ‘Radiation therapists and psychosocial support’ was developed. Items included patient vignettes, embedded items from RT research, and the Professional Quality of Life Scale (ProQOL5). Four radiation oncology departments volunteered to pilot the survey; each nominated four RT staff to participate. Survey data were analysed descriptively and qualitative feedback grouped and coded to determine whether the survey needed to be refined.

**Results:**

Thirteen of sixteen RTs completed the pilot survey and feedback form. Median time to completion was 35 mins, with 54% of respondents stating this was too long. Respondents reported content, questions and response options were relevant and appropriate. Feedback was used to: refine the survey instrument, minimise responder burden and drop out and improve functionality and quality of data collection.

**Conclusion:**

This pilot of the ‘Radiation therapists and psychosocial support’ survey instrument demonstrated content validity and usability. The main survey will be circulated to a representative sample of RTs for completion.

## Introduction

People diagnosed with cancer are likely to experience some psychosocial distress across their cancer trajectory including emotional, social, spiritual and psychological concerns^1^. Both Australian and international statistics reflect the significant issue of psychosocial concerns affecting cancer patients. In Australia, up to 66% of people with cancer experience long‐term psychological distress, and clinically significant anxiety and depression rates have been reported to be 30% and 20–35% respectively^2^. Furthermore, 75% of cancer patients with clinically relevant anxiety and/or depression did not receive counselling or psychological treatment.^3^ These high levels of psychosocial distress and unmet needs in people with cancer have been recognised globally, and have led to the development of psychosocial care guidelines for clinicians in Australia, Canada, United States of America and Europe.^1^, ^2^, ^4^, ^5^, ^6^, ^7^ These guidelines state that psychosocial care involves all health care professionals (HCP) in cancer care,^2^ however, no clear evidence exists demonstrating the implementation of these guidelines by radiation therapists (RTs) into routine practice.

It was estimated that 134,174 Australians would be diagnosed with cancer in 2017^8^ and approximately half could benefit from radiation therapy.^9^ In research conducted in a radiation oncology environment, Mackenzie et al. found that up to 31% of radiation therapy patients reported their care and well‐being could have been improved across two or more patient‐centred care domains of psychosocial care. The most frequently reported categories were: information and communication; emotional and spiritual support; management of physical symptoms; and involvement of friends and family.^10^ Possible reasons for this include lack of multidisciplinary teams, sub‐optimal co‐ordination of care, lack of/or overburdened services, and/or lack of professional knowledge in psychosocial care domains.

RTs are members of the multidisciplinary oncology team responsible for radiation therapy treatment planning and delivery. RT roles include technical and patient care such as communication regarding procedures and technical aspects of treatment, hygiene and self‐care during treatment and appointment scheduling.^11^ As the only members of the radiation oncology team engaging daily with patients throughout their radiation treatment, RTs are uniquely positioned to provide psychosocial support such as patient education and information, or referral to psychosocial services. RTs are motivated to deliver this support according to Hulley who reported 85% of RTs surveyed entered the profession to provide care and emotional support to patients.^12^ Furthermore, Bolderston identified altruism and a desire to help people with cancer, as a common motivator to become an RT.^11^ Despite this high level of motivation, RTs are reported to lack confidence and feel inadequately trained or prepared to discuss psychosocial issues.^11^, ^13^, ^14^, ^15^

Trained professionals with expertise in providing psychosocial care include psychologists, psychiatrists, social workers, counsellors and pastoral carers. We are not proposing RTs fulfil the role of these professionals; rather, RTs are part of the multidisciplinary team who share the responsibility of facilitating holistic care, including psychosocial support. The extent of RT involvement in psychosocial care will vary based on knowledge, confidence, experience, professional networks and local services available and importantly patient needs.

Most research investigating psychosocial support for cancer patients has not included RTs.^16^, ^17^ We conducted a systematic review focused on RT led psychosocial support and its impact on patient anxiety. Of 12 publications identified, three were specific to ‘*Patient Perspectives*’ (qualitative), five to *‘Patient Information/Education*’ and four to *‘Screening and Needs Assessment’*. The review indicated RTs do provide daily supportive care that can reduce anxiety, fear and loneliness. To achieve this, RTs build rapport with patients and provide emotional comfort, information and education.^18^ RT use of screening and needs assessment tools was shown to be feasible and resulted in increased communication and knowledge of patients’ psychosocial concerns.^17^, ^19^, ^20^, ^21^ RT motivation to use screening tools varied, but training appeared to increase RT co‐operation.^18^

Our review summarised current knowledge of RT‐led provision of psychosocial care and identified gaps in the literature.^18^ Four areas requiring further investigation were recognised: RT role clarification; availability and uptake of communication skills training; knowledge of psychosocial referral pathways; and RT involvement in screening and needs assessments.^18^ Consequently, we proposed to conduct an online survey exploring RT perceptions of, and experience in, identifying and managing psychosocial distress. The aims of this paper were to report:

1. the development of an online survey instrument ‘Radiation Therapists and Psychosocial Support Survey’; and
2. the pilot of this instrument with RT professionals – assessing content validity, functionality and length

## Methods

### Survey development

A multidisciplinary research team was formed, including one radiation oncologist, two radiation therapists and one behavioural scientist. An online survey was designed using Qualtrics survey software. Use of an online data collection strategy was selected as it is inexpensive, wide reaching and facilitates complete and accurate data collection.^22^ Survey construction was influenced by published guidelines and tailored to an RT population.^22^, ^23^, ^24^

### Ethics

Ethics approval was granted by the University of Sydney Human Research Ethics Committee (project number 2016/227).

### Survey items

The ‘Radiation Therapists and Psychosocial Support Survey’ instrument comprised 147 items grouped into 11 sections (Table 1). This included items and patient vignettes developed for this study, as well as existing questionnaires. All items were reviewed by the research team to ensure study objectives were met. Items were designed to include qualitative and quantitative response options and careful consideration was given to:

- question and response types
- question and response clarity
- logical grouping and order of topics/questions
- maintaining anonymity and confidentiality
- ensuring sensitivity when asking demographic questions; and
- format/layout

**Table 1:** Development of the ‘Radiation Therapists and Psychosocial Support’ survey with reference to existing literature and evidence gaps

Survey section	Existing literature	Evidence gaps in existing literature
3. Communication Skills Training (CST) (Pre test)	Girgis assessed perceived need for CST in other oncology professionals but did not include RTs^36^ Larsen conducted a single centre Canadian study indicated RTs were interested in further education in communication^15^	RTs perceived need and motivation to undertake CST RT perceptions and relevance of CST to their role Potential barriers to partaking in CST
	Turner identified a lack of CST in HCP groups^4^ Review of CST for HCPs did not identify any studies exploring CST in RTs^16^ Diggens et al. identified Victorian RTs who had completed CST^25^ Lavergne concluded 87% of RTs would like further education in management of anxiety and depression^33^	What training RTs undertake in the area of psychosocial care and communications skills as very limited information exists in current literature. RTs perceived value of such training.
4. Values	Hulley assessed perceived value of RTs providing support to emotional patients as part of the RT role^12^ Professional associations and guidelines outline expectations of cancer HCPs, including RTs, to support patients^15^ Bolderston reported RTs technical skills appear more highly valued in the workplace than caring skills^11^ Multiple authors suggest lack of clarity regarding the perceived role of the RT^20^ Diggens suggests RT perception of their role impacts burnout^25^ Egestad reported patients are receptive to RTs providing psychosocial care^37^	Do RTs value their role in providing supportive care to patients and is this valued by colleagues, management and organisations
5. Patient Anxiety	Multiple authors have reported RTs are motivated to provide psychosocial support^11^, ^12^, ^13^ but lack confidence^13^, ^14^ Multiple authors have reported RTs are more comfortable recognising and managing anxiety than depression^33^ Diggens suggested a relationship between confidence in providing psychosocial support and RT burnout^25^ Oultram reported RTs over estimated patient anxiety and suggested further training was necessary^17^	RTs knowledge of signs and symptoms of anxiety RTs confidence in dealing with anxiety
	Halkett reported 95% of RTs surveyed felt distressed patients require more time for their planning appointment than non‐distressed patients^13^	RTs perception of the impact of patient anxiety on the work environment including self, colleagues, appointment scheduling, safety and accuracy of treatment delivery
	Lavergne reported personal experience with anxiety and depression has a positive impact on comfort when dealing with patients with anxiety and depression^33^	The impact of personal experiences on confidence and knowledge of anxiety, on managing a patient with anxiety
6. Vignettes	Halkett studied video recording of RTs, nurses and two patient interactions attending radiation planning sessions. To assist anxious patients, RTs and nurses used strategies to: explore patients feelings, dedicate more time to patient, acknowledge/validate/reassure patient, refer patient to other professionals, provide other sources of information to patient^13^	RTs abilities to detect and manage patients with anxiety
7. Current Work Practices	Multiple authors concur screening for distress is more effective than relying on clinical judgement alone^5^ Braeken and Mitchell independently concluded RTs are not in agreement that screening is effective^19^, ^20^ Maamoun audited radiation therapy treatment records and did not find any referrals to psychosocial care services annotated by RTs^14^ Larsen reported a median rate of referral to nurse, nutritionist, social worker or other for psychosocial care was 25% compared to literature estimate of 30‐39% in a single centre study^15^ Lavergne reported 78% of RTs agreed screening is important while only 16% report checking screening results weekly. Also, 70% of RTs refer to social workers as a first line of action for distressed patients, suggesting RTs are unaware of other services or how to gauge the most appropriate action^33^ Hulley reported 94% of RTs were aware of psychosocial services and how to access these for patients, 70% had access to patient educational resources regarding psychosocial care, and 45% were aware of resources to improve their own ability to deliver psychosocial care^12^	RTs awareness of departmental screening processes and psychosocial resources. RTs involvement in psychosocial screening processes and referral pathways, including initiating referrals
8. Current Work Resources	Multiple authors have identified or suggested barriers to providing psychosocial care. These include: time, space, staffing, knowledge, training, informational resources, organisational culture^12^, ^15^ Maamoun found RTs with more than ten years experience placed significantly higher importance on identifying supportive care needs of patients than RTs with less experience^14^	Perceived barriers to providing psychosocial support in a larger sample size
10. Work Related Stress	Larsen^15^ report RT involvement in psychosocial care increases job satisfaction and personal accomplishment Diggens^25^ concluded dealing with emotional patients has an unclear impact on depersonalisation and emotional exhaustion Multiple authors report organisational and workload factors are strongly related to workplace stress^7^, ^30^, ^32^, ^34^	RTs use of support services for own health Extent of burnout in RTs and associations with other factors (e.g. hours of direct patient care)

Other survey instrument sections not detailed above were: Section 1 – Participant Information Statement including instructions, ethics and consent; Section 2 – Demographics – Individuals and place of employment; Section 9 – Communication Skills Training (post‐test); Section 11 – Additional Information including free text comments, and requests to receive CPD points, study results and/or to be notified of future research.

#### New items

New items were developed to explore the following knowledge gaps (Table 1): RT role definition (section 3); RT communication skills/training (section 3); RT skill in identifying emotional distress (section 6) and use of screening and needs assessment tools (section 7). RT communication skills have been associated with RT confidence and burnout, therefore items were developed to explore this relationship (section 10).^14^, ^25^

#### Patient vignettes

To assess RT ability to detect and manage patient anxiety, three vignettes based on common presentations of radiation therapy patients with psychosocial needs were developed. Using structured vignettes is an informative approach to assessing skills, and examining factors that influence respondents.^26^ Guided by previous research, the vignettes describe three fictitious patients in a radiation therapy setting, with non‐gender‐specific names and diagnoses to minimise potential gender biases.^26^, ^27^ The vignettes depicted patients with varying levels of anxiety and were followed by these four questions (Table 1, section 6): select appropriate descriptors for each patient, list key indicators leading to the selection of these descriptors, list appropriate management for each case, and indicate appropriateness of psychosocial referral.

#### Existing items and instruments

Permission was obtained to embed the Professional Quality of Life Scale (ProQOL5) and items designed by Hulley in research regarding RT interactions with emotional patients.

ProQOL5 is a freely available instrument assessing burnout in ‘helping professions’. It has been widely used in healthcare groups and demonstrated good construct validity. It consists of 30 items, in three sub‐sections, and uses a five‐point Likert response scale. It generates three scores: (i) compassion satisfaction; (ii) compassion fatigue burn out; and, (iii) compassion fatigue secondary traumatic stress. The reported reliability and validity value for the compassion satisfaction and burnout scales are *α* = 0.88 (*n* = 1130); and *α* = 0.75 (*n* = 976) respectively.^28^

Twenty‐six items designed by Hulley, explored the perceived value of RT interactions with emotional patients, and the perceived availability of resources in the work environment to enable RTs to support emotional patients (Table 1, sections 4 and 8).^12^

### Pilot survey and pilot feedback form

Radiation therapy departments volunteered to participate in the pilot, following a presentation (by the first author) at the New South Wales Radiation Therapy Research Showcase. A representative in each department was asked to invite four RTs to assess survey feasibility by completing the online survey and pilot feedback form. Guidelines suggested that invited RTs include a range of: sex, age, experience, interest in patient care and clinical/non‐clinical responsibilities. An email invitation, survey link and feedback form was forwarded to RTs. The participant information statement was available to participants prior to commencing the survey and outlined the following: the purpose of the study, participation is voluntary, ethics approval details and contacts, consent was implied by survey submission, and contact details for two of the researchers. The feedback form consisted of 12 open‐ended questions to encourage qualitative feedback regarding survey content validity, clarity, internal consistency, appropriateness, intent, length and flow.^29^ The pilot survey and feedback form can be found in Data S1–S2 or requested from the corresponding author.

### Pilot process

Three distinct groups, with expertise in medical radiation sciences, medicine, and/or psychology, assessed content validity, clarity of items and item groupings. These groups offered differing perspectives on the relationship of items to the conceptual domain of the survey, which led to survey refinement.

The first group, pilot respondents, completed the survey and the pilot feedback form. The second group consisted of professional association representatives and academics who provided written feedback regarding content validity and survey relevance to international RTs. Thirdly, the research team formed the panel of experts to finalise survey content, based on feedback from groups 1 and 2.^29^

## Results

Invitations to participate in the pilot were sent to 16 RTs in four radiation therapy departments. These departments included a mix of urban, outer metropolitan, public and private organisations. Thirteen RTs (81% response rate) completed the pilot survey (online) and feedback form (via email). Twelve participants responded within two weeks of the email invitation. A reminder was sent two weeks post‐initial invitation, generating receipt of one further survey and feedback form. Of note, one feedback form was returned incomplete (Table 2). The 81% response rate demonstrated acceptability of the survey concept by the target group. Demographics collected, showed a range of personal and work place characteristics (Table 3). No data were collected from the three non‐responders.

**Table 2:** Pilot feedback questions and response summary

No.	Question^a^	No. of responses			
		Yes	No	DNA	n/a
1	How long did the survey take to complete? (median, range)	35 min (20–50 min)			
2	Is this acceptable?	6	7	–	–
3	Were any of the questions unclear?	2	11	–	–
4	Were any of the response options unclear?	2	11	–	–
5	Were any of the response options not appropriate or relevant?	2	11	–	–
6	Did any of the questions make you feel uncomfortable?	2	11	–	–
7	Did you answer the questions that made you feel uncomfortable?	3	3	4	3
8	Were all sections of the survey clearly explained?	11	0	2	–
9	Are there any questions you would like to see taken out of the survey?	2	9	2	–
10	Are there any questions you would like to add to the survey?	1	11	1	–
11	Do you have any further comments or feedback?	6	5	2	–
12	Are you willing to be contacted via phone to further discuss?	5	4	4	–

DNA, did not answer; n/a, not applicable; one participant did not complete questions 8–12.

a Complete pilot feedback question form can be requested from the corresponding author.

**Table 3:** Pilot survey – respondent demographics

Characteristic	Number (%)
Age (mean, range)	39 (25–54)
Number of years as a qualified RT (mean, range)	16 (1–31)
Sex	
Male	4 (31)
Female	9 (69)
Employment status	
Fulltime	9 (69)
Part time	4 (31)
Current role	
Clinical RT	11 (85)
Research RT	2 (15)
Type of organisation	
Public	7 (54)
Private	6 (46)
No. of RT staff in department (mean, range)	30 (10–50)
No. of linear accelerators in department (mean, range)	3 (2–5)

RT, radiation therapist.

Responses to feedback questions were compiled into four thematic groups: (i) time/survey length; (ii) content; (iii) functionality and (iv) other. The responses were discussed by the research team and consensus reached regarding how to amend the survey instrument (Table 4).

**Table 4:** Pilot feedback – summary of comments provided by respondents

No.	Feedback comment	Domain (T, C, F, O)	Status (A, N)	Reason not actioned
1	Did 20 min then lost responses…started over… 30 min to complete	T/F	A	n/a
2	30 min is acceptable. Reduce scenarios to 2	T/C	A	n/a
	Slightly too long to do at work… but appropriate for enough information to be gathered	T	A	n/a
	15 min	T	A	n/a
	Yes, if organisation support is given	T	–	n/a
	Shorter would be better… but to get the information required this is okay	T	A	n/a
	I believe an acceptable time is 10–15 min	T	A	n/a
3	‘Any of the following aspects of RT affected…’ might need ‘potentially’	C	N	Existing tool
	… I was unsure of whether ‘attendance’ meant face to face training or …online	C	A	n/a
4	Some of them could be more specific	C	N	Not specific
	The ‘not sure’ options could be ‘sometimes’ but then there might be… indecisiveness	C	N	Existing tool
	…a neutral option instead or along with the ‘I don't know’ option.	C	N	Existing tool
5	See above (included in other comments)	C	–	n/a
	The traumatic event ones were strange as I haven't had traumatic event	C	–	n/a
6	DOB	C	A	n/a
9	One scenario less	C	A	n/a
	I feel that all questions were relevant and should not be removed	C	–	n/a
	The case studies were not very useful. I would remove or just have one	C	N	Authors disagreed
10	Suggestions on the most optimal ways of effectively communicating	C	N	Authors disagreed
11	Great layout and very comprehensive	C	–	n/a
	…the second scenario story was on the previous page to the question, I went to go back and it went to the beginning of the survey and lost all my answers	F	A	n/a
	No back button on the survey	F	A	n/a
	… contained appropriate questions and answers however…a bit lengthy	T	A	n/a
	Fantastic	O	–	n/a
	This is an important topic and happy to contribute	O	–	n/a

n/a, not applicable; T, time; F, functionality; C, content; O, other; A, actioned; N, not actioned.

*i) Time/Survey Length*

Seven of 13 respondents reported time to complete the survey was too long (median 35 min, range 20–50 min). To reduce the survey length and respondent burden, the following items were removed:

- one item (2.8) requesting postcode of the radiation therapy department;
- two post‐vignette items (9.2 and 9.3 repeated before and after the vignettes) related to the ‘perceived need and motivation for communication skills training’;
- one vignette and related items (6.12–6.16) as suggested by two respondents. One respondent suggested removing all vignettes; however, based on overall feedback and research team preference, two vignettes were retained.

*ii) Content*

Survey questions and response options were clear, appropriate and relevant according to 11 of 13 respondents. Three comments suggested neutral response options to three items (4.2, 8.2 and 8.4) would be preferable. These were existing items from survey tools, hence this change was not made, as the research team wanted to ensure comparability of results with previous studies.

One respondent noted requesting date of birth (DOB) may deter RTs from completing the survey. Researchers felt DOB was useful to enable more accurate data reporting and that participants were not at risk of being identified due to confidentiality protocols and the large sample size of the main survey. Consequently, DOB was changed to a non‐mandatory field.

Following feedback from one respondent, one item (3.7) regarding training in the area of patient care, was reworded to include both face‐to‐face and online training.

*iii) Functionality*

Three respondents highlighted the absence of a ‘back’ button to view previous information. Therefore, a ‘back’ button was added.

*iv) Other*

Two respondents provided positive comments relating to the survey and research concept.

### Interviews

The pilot feedback form asked participants to provide consent and contact details if they were willing to be contacted for an interview. Five participants provided these details, however, the research team decided not to conduct interviews as the feedback was clear and consistent, and no further information would be gained.

### Additional Information

In addition to the pilot process, three medical radiation professionals (identified by professional medical radiation associations in Australia, New Zealand (NZ) and Canada) as well as one medical radiation academic reviewed the survey instrument and provided written feedback. The survey was sent to these reviewers as a word document via email and feedback was returned to the first author. This process was conducted after the pilot. This feedback was supportive and highlighted the value of this research. The concerns raised were survey length and the sensitive nature of some questions. To address this, further modifications were made to reduce the number of items relating to each vignette, and open‐ended questions were changed to questions with multiple choice response options. Feedback regarding DOB was similar to comments made by a pilot respondent, thus confirming the decision to make DOB non‐mandatory. Additional modifications included a ‘Prefer not to answer’ response option for items regarding: carer responsibilities, year RT commenced practice, and personal experience with anxiety.

## Discussion

An online survey was selected as an effective method to explore RT values, skills, training and knowledge regarding psychosocial support for patients undergoing radiation therapy. An online survey is an inexpensive, wide reaching approach, which enables collation and analysis of large volumes of data in a short timeframe.^22^ Other multicentre surveys targeting RTs have yielded encouraging response rates in Australia, NZ and Canada, of 37–41%, 48% and 21–36% respectively^7^, ^12^, ^14^, ^30^, ^31^, ^32^, ^33^, ^34^.

The ‘RTs and Psychosocial Support Survey’ instrument was developed to address gaps in the literature regarding provision of psychosocial support in the radiation oncology setting^18^ (Table 1). Existing items were embedded and literature‐guided survey development to ensure data could be compared.

Piloting a survey is an important component of the development, feasibility and evaluation process.^23^, ^24^, ^35^ The pilot was conducted to test the acceptability and suitability of the online survey and recruitment process among a convenience sample of RTs. RTs provided valuable information on content validity, face validity, length of survey and functionality. An 81% response rate and positive feedback indicated strong support for the survey and psychosocial care research in radiation therapy. This feedback was reinforced by professional associations who agreed to support, circulate and promote the main survey to their membership.

The pilot study resulted in a more concise survey instrument, with increased likelihood of completion by busy RTs. This survey instrument is potentially applicable in multiple radiation oncology departments globally, to assess RT values, skills, training and knowledge specific to detecting and managing patient anxiety. This instrument provides a contribution to the field of radiation therapy^22^ that may be used by others in future research, with potential to improve the delivery of psychosocial care and reduce the number of patients with unmet psychosocial needs.

Following the pilot and instrument refinement, the main ‘Radiation Therapists and Psychosocial Support Survey’ was launched via Qualtrics to RTs in Australia, NZ and Canada. These countries formed the target demographic due to similarities in training, workforce and clinical practice. Publications related to RTs, psychosocial support and burnout produced by these countries further strengthened the decision to invite them to participate. Data yielded from this survey will be compared to existing literature to test generalisability across a larger sample. Results of the main survey will be detailed in future publications.

There were limitations to this study. First, surveys are susceptible to responder bias and we did not collect demographics of non‐responders, or reason for not responding. Second, the survey was estimated to take 30 mins to complete and requested sensitive information. These factors may have led to RTs not completing the pilot survey. Lastly, all pilot participants were recruited from one Australian state. To address this issue of convenience sampling and assess survey content validity for a wider audience, the pilot survey was reviewed by local and international academics and professional associations.

## Conclusion

Piloting the online survey instrument was informative. Feedback provided by participating RTs resulted in modifications to reduce survey length, clarify content and increase functionality of the instrument. The pilot process resulted in a refined survey instrument, which will minimise responder burden and drop out, and improve the likelihood of obtaining a representative sample of RTs in the main survey. These results demonstrate that the ‘Radiation Therapists and Psychosocial Support Survey’ is a useable instrument likely to yield informative results in exploring RTs values, skills, training and knowledge regarding patient anxiety and psychosocial support.

## Conflict of Interest

The authors declare no conflict of interest.

## Supporting information


**Data S1:** Pilot Survey Questions.

**Data S2:** Feedback Questions.
