# Supplementary material for: Development and pilot of an international survey: ‘Radiation Therapists and Psychosocial Support’
Source: J Med Radiat Sci. 2018 Jun 7;65(3):209–17. doi: 10.1002/jmrs.286 (PMC6119728; doi:10.1002/jmrs.286)
Supplement: Supplementary file 1 — Data S1: Pilot Survey Questions. [file JMRS-65-209-s001.docx]

**Supporting Information**

**Pilot Survey Questions**

Question 1 was the Participant Information Statement and has not been included here. The authors will provide this information on request.

Q2.1 What is your sex?

- Male (1)
- Female (2)

Q2.2 What is your date of birth? (Please enter in the following format dd/mm/yyyy).

Q2.3 Do you have carer responsibilities in your personal life and if so what are they? Select all that apply.

- Yes, children (1)
- Yes, elderly family member or friend (2)
- Yes, unwell family member or friend (3)
- Yes, disabled family member or friend (4)
- Yes, other (5)
- No (6)

Q2.4 What year did you start work as a Radiation Therapist? (Please enter in the format yyyy). (Note: For some Australian RTs, this is the year you started your SPP/NPDP/PDY)

Q2.5 What is your current employment status?

- Full time (1)
- Part time (2)
- Casual (3)
- Not currently employed (4)
- Retired (5)
- Other, please specify below (e.g. on extended leave) (6) ____________________

Q2.6 Please answer the following questions with reference to the last radiation therapy job you had.

Q2.7 Which country are you working in?

- Australia (1)
- New Zealand (2)
- Canada (3)

Q2.8 What is the postcode of your work place?

Q2.9 What is your primary professional role?

- Clinical Radiation Therapist (1)
- Radiation Therapy Educator (4)
- Research Radiation Therapist (5)
- Manager (2)
- Other, please specify below (3) ____________________

Q2.10 What type of radiation oncology organisation do you work in?

- Public centre (1)
- Private centre (2)
- Other, please specify (3) ____________________

Q2.11 How many hours of direct patient care do you engage in per week, within your current role? (This will vary depending on your current rostered area i.e. planning or treatment.)

- None (1)
- 1-10 hours (2)
- 11 - 30 hours (3)
- More than 30 hours (4)

Q2.12 How many radiation therapy staff work in your department? (If unsure, please estimate)

Q2.13 How many linear accelerators (linac) are used to treat patients at your place of work?

Q2.14 How many radiation therapists are rostered to a linac on a usual day of operation?

Q2.15 How many patients are treated on a linac on a usual day of operation? Please estimate.

Q2.16 How many hours per day is the linac operating to treat patients? e.g. 8 hours

Q3.1 Communication Skills Training programs are designed for Health Care Professions (HCPs) to develop skills which enable them to build positive relationships with patients diagnosed with a long term illness. These programs focus on skills such as appropriate verbal communications, responding to non-verbal cues, active listening and expressing empathy. HCPs may participate in role plays with actors, who simulate patients by acting out challenging scenarios inspired by real life cases. Examples of cases may include patients with anxiety, depression, aggressive behaviour or distress.

Q3.2 How strongly do you rate your need for Communication Skills Training?

- Strong (1)
- Moderate (2)
- Low (3)
- None (4)

Q3.3 How strongly do you rate your motivation to complete Communication Skills Training?

- Strong (1)
- Moderate (2)
- Low (3)
- None (4)

Q3.4 How do you think Communication Skills Training would affect you and your work?

|  | Positive change (1) | Negative change (2) | No change (3) |
| --- | --- | --- | --- |
| Confidence in dealing with patients (1) |  |  |  |
| Efficiency in dealing with patients (2) |  |  |  |
| Providing emotional support for patients (3) |  |  |  |
| Providing emotional support for patients' carers and significant others (4) |  |  |  |
| Showing respect for patients (5) |  |  |  |
| Approachability (i.e. the patient's openness to approaching you for information) (6) |  |  |  |
| Providing information to patients (7) |  |  |  |
| Work related stress (8) |  |  |  |
| Personal well-being (9) |  |  |  |
| Job satisfaction (10) |  |  |  |
| Providing support for colleagues (11) |  |  |  |

Q3.5 Are there other ways Communication Skills Training may affect you and your work?

- Yes (1)
- No (2)

Q3.6 If yes, please specify.

Q3.7 Please indicate if and when you have participated in training in the following areas of patient care.

|  | Attended | | Timeframe | | | |
| --- | --- | --- | --- | --- | --- | --- |
|  | Yes (1) | No (2) | Less than 1 year ago (1) | Between 1 and 5 years ago (2) | More than 5 years ago (3) | N/A (4) |
| Communication Skills Training (1) |  |  |  |  |  |  |
| Detecting and responding to emotional cues (2) |  |  |  |  |  |  |
| Patient psychology (3) |  |  |  |  |  |  |
| Patient counselling (4) |  |  |  |  |  |  |
| Patient anxiety and depression (5) |  |  |  |  |  |  |

Q3.8 Are there other areas of patient care training that you have participated in?

- Yes (1)
- No (2)

Q3.9 If yes, please specify details including name of training/course, focus of training/course and approximate year attended.

Q3.10 What would stop you from accessing Communication Skills Training? Select all that apply.

- Interest (1)
- Course costs (2)
- Location of courses (3)
- Personal time (4)
- Personal energy (5)
- Release time from work to attend training (6)
- Managerial support (7)
- Organisational support (8)
- Co-worker perceptions (9)

Q3.11 Are there any other issues that would stop you accessing Communication Skills Training?

- Yes (1)
- No (2)

Q3.12 If yes, please specify.

Q4.1 We are interested in the "value" of radiation therapists' abilities to interact with emotional patients. The following questions refer to "emotional" patients and "psychosocial" issues. These terms have been defined here for clarity. An "emotional" patient is one who is markedly aroused or agitated in feeling or sensibilities. An "emotional patient" may display signs of crying, not coping well, being at a loss for words, being agitated, upset, or angry. "Psychosocial" refers to both psychological and social behaviour. Assessing the psychosocial well-being of a patient relates to noticing how the patient is coping with respect to their mental, emotional, social and spiritual well-being. Please keep these definitions of "emotional patient" and "psychosocial" in mind for the remainder of the survey.

Q4.2 For the statements below, please indicate the response that best matches your opinion (Agree, Disagree, or Don't know)

|  | Agree (1) | Disagree (2) | Don't know (3) |
| --- | --- | --- | --- |
| Patient care and support is the main reason I entered this profession (1) |  |  |  |
| My organisation (e.g. hospital, clinic) values my providing support to emotional patients (2) |  |  |  |
| My work colleagues (other radiation therapists) value my providing support to emotional patients (3) |  |  |  |
| Other members of the health care team value my providing support to emotional patients (4) |  |  |  |
| At my workplace it is expected that radiation therapists provide support to emotional patients (5) |  |  |  |
| The focus of radiation therapy should be on technical innovations rather than patient care (6) |  |  |  |
| Emphasis on technical skills is driving recruitment and retention processes for radiation therapists (7) |  |  |  |
| My patients feel questions about the psychosocial aspects of their lives are irrelevant (8) |  |  |  |
| A patient's family will reject the idea of radiation therapists dealing with psychosocial issues (9) |  |  |  |
| Patients are not open to radiation therapists dealing with psychosocial issues (10) |  |  |  |

Q5.1 Being distant is often recognised as a sign of depression. Can you think of recognisable signs of ANXIETY? Please list 3 or more.

Q5.2 How confident do you feel when dealing with a patient showing signs of anxiety?

- Very Confident (1)
- Somewhat confident (2)
- A little confident (3)
- Not at all confident (4)

Q5.3 Are any of the following aspects of radiation therapy affected when treating an anxious patient?

|  | Yes (1) | No (2) |
| --- | --- | --- |
| Time to manage the patient (1) |  |  |
| Time to treat the patient (2) |  |  |
| Safe treatment delivery (3) |  |  |
| Accurate treatment delivery (4) |  |  |
| Staffing levels (5) |  |  |
| Daily appointment schedule (6) |  |  |
| My emotional state (7) |  |  |
| Emotional state of fellow RTs (8) |  |  |
| My work related stress levels (9) |  |  |
| My job satisfaction (10) |  |  |
| My confidence (11) |  |  |

Q5.4 Are any other aspects of radiation therapy affected?

- Yes (1)
- No (2)

Q5.5 If yes, please specify.

Q5.6 Have you been involved in detecting and dealing with anxiety outside of your professional work? That is, in yourself, or others in your personal life.

- Yes (1)
- No (2)

Q5.7 If yes, who was the person experiencing anxiety? Select all that apply.

- Myself (1)
- A family member (2)
- A close friend (3)
- A partner (4)
- Other (5)
- Prefer not to answer (6)

Q6.1 For the following three vignettes, read the case and then answer the questions regarding the patient presented. Answer honestly and note that "I don't know" is an acceptable answer.

Q6.2 Vignette 1 – Alex

Alex is 63 years old and has been diagnosed with metastatic lung cancer. The Radiation Oncologist has informed Alex of a poor prognosis and recommended a palliative radiation therapy treatment regimen. Alex presents for the first day of radiation therapy accompanied by a friend. In the waiting room Alex is sitting straight and rigid, with hands clasped holding a tissue and appears a bit tearful. Alex is quiet but attentive, nodding in response to information you give, although you do notice that Alex has asked you to repeat information a couple of times.  Alex asks some questions about radiation damage to the body and is then willing to proceed with treatment.

Q6.3 Which of the following descriptions would you apply to Alex? Select all that apply.

- Depressed (11)
- Happy (1)
- Angry (2)
- Calm (3)
- Sad (4)
- Cheerful (5)
- Distressed (6)
- Worried (7)
- Relaxed (9)
- Anxious (8)
- I don't know (10)

Q6.4 What signals led you to select these responses?

Q6.5 What would you do to help Alex?

Q6.6 Do you think a referral to a psychosocial care giver would be appropriate for Alex?

- Yes (1)
- Maybe (2)
- No (3)
- I don't know (4)

Q6.7 Vignette 2 - Pat

Pat is 46 years old, has been diagnosed with lymphoma and is aware of the likelihood of a good prognosis. The Radiation Oncologist has recommended a curative radiation therapy treatment regimen. Pat arrives alone for the radiation therapy planning session (CT simulation) and appears rushed and agitated. Pat asks many questions about simulation and the planned treatment. Pat  does not appear to be listening to your responses and keeps asking questions, sounding increasingly annoyed. Pat has many complaints about having to attend radiotherapy and does not believe that the appointment times will fit into daily life. Pat does not wish to have tattoos and demands to see the doctor before agreeing to proceed with the session.

Q6.8 Which of the following descriptions would you apply to Pat? Select all that apply.

- Depressed (11)
- Happy (1)
- Angry (2)
- Calm (3)
- Sad (4)
- Cheerful (5)
- Distressed (6)
- Worried (7)
- Relaxed (9)
- Anxious (8)
- I don't know (10)

Q6.9 What signals led you to select these responses?

Q6.10 What would you do to help Pat?

Q6.11 Do you think a referral to a psychosocial care giver would be appropriate for Pat?

- Yes (1)
- Maybe (2)
- No (3)
- I don't know (4)

Q6.12 Vignette 3 - Taylor

Taylor is 24 years old and has been diagnosed with a sarcoma. The Radiation Oncologist informed Taylor of a possibly poor prognosis but recommended a curative radiation therapy treatment regimen. Taylor and Taylor’s mother arrive for the first radiation therapy treatment. Taylor is fiddling with car keys and reports concern about being able to lie still for treatment, not being able to sleep last night and feeling nauseous.

Q6.13 Which of the following descriptions would you apply to Taylor? Select all that apply.

- Depressed (11)
- Happy (1)
- Angry (2)
- Calm (3)
- Sad (4)
- Cheerful (5)
- Distressed (6)
- Worried (7)
- Relaxed (9)
- Anxious (8)
- I don't know (10)

Q6.14 What signals led you to select these responses?

Q6.15 What would you do to help Taylor?

Q6.16 Do you think a referral to a psychosocial care giver would be appropriate for Taylor?

- Yes (1)
- Maybe (2)
- No (3)
- I don't know (4)

Q7.1 The following questions refer to your current workplace practices. They explore processes of screening patients for anxiety, depression and/or distress, available psychosocial support services and processes of referring patients.

Q7.2 Does your workplace have access to the following psychosocial support services? Select all that apply.

- Psycho-oncology (1)
- Social work (2)
- Counselling (3)
- Psychology (4)
- Other, please specify below (5) ____________________

Q7.3 In your workplace, are patients formally screened for anxiety, depression and/or distress?

- Yes (1)
- No (2)
- I don't know (3)

Q7.4 Who routinely does the screening? Select one option only.

- Radiation Oncology Consultant or Trainee (1)
- Radiation Therapist (4)
- Radiation Nurse (5)
- Administrative staff (7)
- Other, please specify below (6) ____________________

Q7.5 Are screening results communicated to you?

- Always (15)
- Most of the time (16)
- Rarely (17)
- Never (18)

Q7.6 How are the results communicated? Select all that apply.

- Written communication (e.g. Radiation therapy information systems e.g.,. ARIA, MOSAIQ; inpatient notes or email) (1)
- Verbal communication (2)
- Other, please specify below (4) ____________________

Q7.7 Do screening results affect how you approach the patient?

- Always (16)
- Most of the time (17)
- Rarely (18)
- Never (3)

Q7.8 Do you personally refer patients to psychosocial support services?

- Yes (1)
- No (2)

Q7.9 Do you communicate your referral to others?

- Always (22)
- Most of the time (23)
- Rarely (24)
- Never (25)

Q7.10 Who do you communicate the referral to? Select all that apply.

- Patient (1)
- Patient's family or carer (2)
- Radiation Oncology Consultant or Trainee (3)
- Other Radiation Therapists (4)
- Radiation Nurse (5)
- Other, please specify below (6) ____________________

Q7.11 How do you communicate the referral? Select all that apply.

- Written communication (e.g. Radiation therapy information systems e.g.,. ARIA, MOSAIQ; inpatient notes or email) (1)
- Verbal communication (2)
- Other, please specify below (3) ____________________

Q8.1 Sometimes the environment in which we work, plays a role in our ability to interact with emotional patients. This can include the availability of physical, educational and supportive resources.

Q8.2 For the statements below, indicate the response that best matches your opinion.

|  | Agree (1) | Disagree (2) | I don't know (3) |
| --- | --- | --- | --- |
| I am too busy at work to provide support to emotional patients (1) |  |  |  |
| There is no time in the schedule to provide support to emotional patients (2) |  |  |  |
| I often have to prioritise technical demands over support to emotional patients (3) |  |  |  |
| Increasing treatment complexity has reduced the time available to provide support to emotional patients (4) |  |  |  |
| I am too exhausted to provide support to emotional patients (5) |  |  |  |

Q8.3 Consider your current workplace practices when providing answers to the following statement. Select all that apply."When confronted with an emotional patient, I am able to......."

- Take them to a private room to talk (1)
- Talk with them inside the treatment room or control area (2)
- Call on another colleague to cover me while I speak with the patient (3)
- Refer the patient to someone else they can talk with (4)
- Other, please specify below (5) ____________________

Q8.4 For the statements below, indicate the response that best matches your opinion.

|  | Yes (1) | No (2) | I don't know (3) |
| --- | --- | --- | --- |
| I have enough time to fully communicate with emotional patients (1) |  |  |  |
| There are sufficient radiation therapists to take over my duties while I communicate with an emotional patient (2) |  |  |  |
| There are support services I can access to help an emotional patient (3) |  |  |  |
| I am aware of how to access support services to help an emotional patient (4) |  |  |  |
| I can go to my manager or supervisor for support when I care for an emotional patient (5) |  |  |  |
| I can go to the patient's radiation oncologist for support when I care for an emotional patient (6) |  |  |  |
| I have access to patient education materials to help support emotional patients (7) |  |  |  |
| There are educational resources available to me to improve my ability to communicate with emotional patients (8) |  |  |  |
| There are supportive resources available to me to help me deal with providing support to emotional patients (9) |  |  |  |
| My team supports me emotionally when I deal with emotional patients (10) |  |  |  |

Q9.1 Communication Skills Training programs (CST) are designed for Health Care Professionals (HCPs) to develop skills that enable them to build positive relationships with patients diagnosed with a long term illness. You have already answered some questions regarding CST. Please answer the following questions quickly, without dwelling on your responses.

Q9.2 How strongly do you rate your need to complete Communication Skills Training?

- Strong (1)
- Moderate (2)
- Low (3)
- None (4)

Q9.3 How strongly do you rate your motivation to complete Communication Skills Training?

- Strong (1)
- Moderate (2)
- Low (3)
- None (4)

Q10.1 The following questions ask about your feelings and methods of coping with work related issues.

Q10.2 Have you ever used services to help deal with work related stresses of any kind? Types of services may include employee assistance programs, counsellor, debriefing services, G.P., psychologist, mental health services, etc.

- Yes (1)
- No (2)
- Prefer not to answer (3)

Q10.3 Please approximate when you last accessed these services.

- Within the last 12 months (1)
- 1 to 5 years ago (2)
- More than 5 years ago (3)
- Prefer not to answer (4)

Q10.4 As a radiation therapist, being a "helper" and assisting patients and families cope with cancer is part of your role. When you help people, you have direct contact with their lives. As you may have found, your compassion for those you help can affect you in positive and negative ways. Below are some questions about your experiences, both positive and negative, as a "helper". Consider each of the following questions about you and your current work situation. Select the option that honestly reflects how frequently you have experienced these things in the last 30 days.

|  | Very Often (1) | Often (2) | Sometimes (3) | Rarely (4) | Never (5) |
| --- | --- | --- | --- | --- | --- |
| I am happy (1) |  |  |  |  |  |
| I am preoccupied with more than one person I help (2) |  |  |  |  |  |
| I get satisfaction from being able to help people (3) |  |  |  |  |  |
| I feel connected to others (4) |  |  |  |  |  |
| I jump or am startled by unexpected sounds (5) |  |  |  |  |  |
| I feel invigorated after working with those I help (6) |  |  |  |  |  |
| I find it difficult to separate my personal life from my life as a helper (7) |  |  |  |  |  |
| I am not as productive at work because I am losing sleep over traumatic experiences of a person I help (8) |  |  |  |  |  |
| I think that I might have been affected by the traumatic stress of those I help (9) |  |  |  |  |  |
| I feel trapped by my job as a helper (10) |  |  |  |  |  |
| Because of my helping I have felt "on edge" about various things (11) |  |  |  |  |  |
| I like my work as a helper (12) |  |  |  |  |  |
| I feel depressed because of the traumatic experiences of the people I help (13) |  |  |  |  |  |
| I feel as though I am experiencing the trauma of someone I have helped (14) |  |  |  |  |  |
| I have beliefs that sustain me (15) |  |  |  |  |  |
| I am pleased how I am able to keep up with helping techniques and protocols (16) |  |  |  |  |  |
| I am the person I always wanted to be (17) |  |  |  |  |  |
| My work makes me feel satisfied (18) |  |  |  |  |  |
| I feel worn out because of my work as a helper (19) |  |  |  |  |  |
| I have happy thoughts and feelings about those I help and how I could help them (20) |  |  |  |  |  |
| I feel overwhelmed because my work load seems endless (21) |  |  |  |  |  |
| I believe I can make a difference through my work (22) |  |  |  |  |  |
| I avoid certain activities or situations because they remind me of frightening experiences of the people I help (23) |  |  |  |  |  |
| I am proud of what I can do to help (24) |  |  |  |  |  |
| As a result of my helping I have intrusive, frightening thoughts (25) |  |  |  |  |  |
| I feel "bogged down" by the system (26) |  |  |  |  |  |
| I have thoughts that I am a "success" as a helper (27) |  |  |  |  |  |
| I can't recall important parts of my work with trauma victims (28) |  |  |  |  |  |
| I am a very caring person (29) |  |  |  |  |  |
| I am happy that I chose to do this work (30) |  |  |  |  |  |

Q10.5 If these questions have raised concerns and you feel you may be suffering stress or burnout, please contact local services for support such as: your manager, employee assistance program representative, G.P., professional body, Lifeline or other group.

Q11.1 Do you have any other comments you would like to add?

- Yes (1)
- No (2)

Q11.2 If yes, please specify.

Q11.3 Please remember, your survey responses will remain anonymous. Any details that you provide in the following questions will remain confidential. Your contact information will be extracted and stored separately to your survey responses.

Q11.4 What further involvement would you like to have after this survey? (Select all that apply)

- I want to claim CPD points for survey completion (1)
- I would like to receive the results of this study once completed (2)
- I am willing to be contacted in the future, to consider taking part in further work that results from this study (3)
- I do not wish to have any further involvement (4)

Q11.5 Please enter your email address to receive an email to support your claim for CPD points

Q11.6 Please enter your email address to receive results of this study

Q11.7 Please enter the following details so that we may invite you to participate in further work resulting from this study.

First name (1)

Last name (2)

Phone number (AUS) (3)

Email address (11)

Q11.8 Please enter the following details so that we may invite you to participate in further work resulting from this study.

First name (1)

Last name (2)

Phone number (NZ) (3)

Email address (11)

Q11.9 Please enter the following details so that we may invite you to participate in further work resulting from this study.

First name (1)

Last name (2)

Phone number (CA) (3)

Email address (11)
