# Supplementary material for: Development and pilot of an international survey: ‘Radiation Therapists and Psychosocial Support’
Source: J Med Radiat Sci. 2018 Jun 7;65(3):209–17. doi: 10.1002/jmrs.286 (PMC6119728; doi:10.1002/jmrs.286)
Supplement: Supplementary file 2 — Data S2: Feedback Questions. [file JMRS-65-209-s002.docx]

**Supporting Information**

**Feedback Questions**

1. How long did the survey take to complete?
2. Is this an acceptable time? If no, what is an acceptable time?
3. Were any of the questions unclear? If yes, please specify the question and explain the issue.
4. Were any of the response options unclear? If yes, please specify the response and explain the issue.
5. Were any of the response options not appropriate or relevant? If yes, please specify the response and explain the issue.
6. Did any of the questions make you feel uncomfortable? If yes, please specify the question and explain the issue.
7. Did you answer the questions that made you feel uncomfortable?
8. Were all sections of the survey clearly explained? If not, please specify the section and explain the issue.
9. Are there any questions you would like to see taken out of the survey? If yes, please specify the question.
10. Are there any questions you would like to add to the survey? If yes, please specify the question and possible response options.
11. Do you have any further comments or feedback?
12. Are you willing to be contacted via phone to further discuss your responses to the feedback questions above. If so, please provide and contact name and phone number.
